# Acupuncture for autophagy in animal models of middle cerebral artery occlusion: A systematic review and meta-analysis protocol

**DOI:** 10.1371/journal.pone.0281956

**Published:** 2023-02-22

**Authors:** Jingxue Yuan, Ziniu Zhang, Jinxia Ni, Xiaona Wu, Haoyue Yan, Jingni Xu, Qi Zhao, Hongwei Yuan, Lei Yang

**Affiliations:** Department of Acupuncture, Dongzhimen Hospital, Beijing University of Chinese Medicine, Beijing, China; GITAM Institute of Pharmacy, INDIA

## Abstract

**Introduction:**

Ischemic stroke has high morbidity, disability and mortality rates. The effective treatments recommended by guideline have considerable limitations due to their strict range of adaptation and narrow time window. Acupuncture is an effective and safe treatment for ischemic stroke, and the mechanism may be related to autophagy. In this systematic review, we aim to summarize and evaluate the evidence of autophagy in acupuncture therapy for animal models of middle cerebral artery occlusion (MCAO).

**Methods:**

Publications will be retrieved from the MEDLINE, Embase, Cochrane Library, Web of Science, CNKI, CBM, CVIP and Wanfang databases. We will include animal experimental studies of acupuncture for MCAO, and the control group will receive placebo/sham acupuncture or no treatment after model establishment. Outcome measures must include autophagy and will include neurologic scores and/or infarct size. The Systematic Review Center for Laboratory animal Experimentation (SYRCLE) risk of bias tool will be used to assess the risk of bias. A meta-analysis will be performed if the included studies are sufficiently homogenous. Subgroup analyses will be conducted according to different intervention types and different types of outcomes. Sensitivity analyses will also performed to explore the heterogeneity and to assess the stability of the results. Publication bias will be assessed by funnel plots. The Grading of Recommendations, Assessment, Development, and Evaluation (GRADE) system will be applied to evaluate the quality of evidence in this systematic review.

**Discussion:**

The results of this study may help to explain autophagy in acupuncture therapy for ischemic stroke. The limitation of this review is that all included studies will be retrieved from Chinese or English medical databases due to language barriers.

**Registration:**

We registered in PROSPERO on May 31, 2022. (https://www.crd.york.ac.uk/PROSPERO/display_record.php?RecordID=329917) (CRD42022329917).

## Introduction

### Description of the disease and autophagy

Stroke is a central nervous system disease with high morbidity, disability rate and mortality. In the most recent Global Burden of Disease (GBD) Study, there were 12.2 million incident strokes, 101 million prevalent strokes, 143 million disability-adjusted life-years (DALYs) due to stroke, and 6.55 million deaths from stroke in 2019. Stroke was the second-leading Level 3 cause of death (classified by GBD) and was also the third-leading Level 3 cause of death and disability combined [1]. Pathological subtypes of stroke comprise ischemic stroke and hemorrhagic stroke [2]. Ischemic stroke accounted for 62.4% of all strokes in 2019 [1], remaining the most frequently occurring type of stroke.

Ischemic brain tissues can be grouped into irreversibly damaged infarct core and reversibly injured ischemic penumbra [3]. The treatment purpose of acute ischemic stroke is to salvage penumbral tissue and improve brain function [4]. Reperfusion that takes place in the reopening of arterial vessels may also cause injury. Disturbed blood flow regulation in the penumbra and lower areas may cause a complex cascade, including excitotoxicity, mitochondrial death pathways, the release of free radicals, protein misfolding, apoptosis, necrosis, autophagy and inflammation [5, 6]. They work alone or in combination in the disease, playing beneficial, deleterious or dual roles in the progression of poststroke brain injury [6]. These may be potential therapeutic targets for ischemic stroke.

Autophagy is a major pathway for eukaryotes to identify and recycle unwanted or dysfunctional components by engaging sophisticated catabolic pathways and maintaining internal homeostasis [7]. Autophagy is divided into two categories: selective autophagy and nonselective autophagy. Selective autophagy refers to the degradation of a specific substrate, such as mitophagy, reticulophagy and ribophagy. Nonselective autophagy can be divided into three main types–macroautophagy, microautophagy, and chaperone-mediated autophagy–based on how intracellular substrates are transported to lysosomes. Different types of autophagy have unique molecular machineries and are tightly regulated by various cellular signals [8]. The existence of and changes in autophagy can be observed by certain submicroscopic structures (e.g., autophagosomes and autolysosomes) in cells or biomarkers (e.g., LC3 and p62) with their specific significance.

Autophagy may be involved in neuroprotective mechanisms after ischemic stroke. Studies have shown that interventions can play a neuroprotective role by regulating autophagy [9, 10]. However, it has not been determined whether this effect occurs through enhancing or inhibiting autophagy [11].

### Description of the model

Occlusion of the middle cerebral artery (MCA), or a branch of it, is the most commonly identified type of human ischemic stroke, which makes the MCA the most common target for animal models [12]. Several middle cerebral artery occlusion (MCAO) models of focal cerebral ischemia have been developed, such as intraluminal filament MCAO, electrocoagulation MCAO, endothelin-1 MCAO, clip/mechanical device MCAO, autologous blood clot MCAO, MCA branch occlusion by intravascular thrombin injection and photochemical ischemia [13, 14]. All models have advantages and disadvantages, and models will be selected based on different experiments [13].

### Description of the intervention

Acupuncture therapy, which originated in ancient China, is a method to treat or prevent diseases by stimulating acupoints and meridians with interventions (e.g., acupuncture, electroacupuncture and moxibustion) under the guidance of basic theories of traditional Chinese medicine [15]. Recently, modern medicine has been more willing to explain the physiological effects and clinical responses of acupuncture therapy from aspects such as neural hypothesis and neural pathways activated by acupuncture [16]. Since 2002, stroke has been listed by the World Health Organization (WHO) as one of the diseases for which acupuncture has been proven to be effective through controlled trials [17]. A recent review also shows that acupuncture therapy is widely used in diseases including poststroke aphasia [15].

### How the intervention might work

Acupuncture, as a nonpharmacological therapy, has high safety and may provide a new opportunity for the treatment of ischemic stroke [18]. A meta-analysis showed that the Xingnao Kaiqiao needling method might significantly reduce the disability rate, improve clinical efficacy, and enhance patients’ activities of daily living (Barthel index), but it had no significant impact on mortality [19]. Another meta-analysis reported that scalp acupuncture therapy can improve neurological deficit scores and the clinical effective rate in patients with acute ischemic stroke [20].

The mechanism of acupuncture for ischemic stroke may be related to the fact that acupuncture therapy can generate beneficial signals to the brain through peripheral nerve pathways. These signals regulate cerebral blood flow and balance survival and death signals at the cellular and molecular levels [18]. Autophagy is one of the neuroprotective mechanisms, and acupuncture therapy regulates autophagy to play a neuroprotective role [21]. Studies have shown that electroacupuncture produces neuroprotective effects by inhibiting neuronal autophagy [22, 23]. It has also been shown that this neuroprotective effect is achieved through the upregulation of autophagy [24, 25].

### The importance and aim of the review

Intravenous thrombolysis and mechanical thrombectomy are two treatments recommended by the guideline for acute ischemic stroke, but they have considerable limitations due to their strict adaptive range and narrow time window [26]. Therefore, it is important to find safe and effective intervention methods after cerebral ischemia. Acupuncture therapy shows promising research prospects, as we mentioned above, but the mechanisms of its effect are not clear.

Preclinical animal experiments can increase our understanding of stroke pathophysiology and possible mechanisms, helping us advance research [27]. To our knowledge, no systematic review or meta-analysis has examined the autophagy mechanism of acupuncture therapy for ischemic stroke in animal models. We will conduct a preclinical systematic review to summarize and evaluate the evidence of autophagy in acupuncture therapy for ischemic stroke.

### Research questions

What is the effect of acupuncture therapy on autophagy in focal cerebral infarction animal models of MCAO?

## Methods

### Search methods for the identification of studies

We will search the following electronic bibliographic databases: MEDLINE, Embase, Cochrane Library, Web of Science, CNKI, CBM, CVIP and Wanfang. We will also conduct online search or attempt to contact the authors for collecting gray literature. The full search strategy (Table 1) is based on the search components “brain ischemia”, “animals, laboratory”, “acupuncture” and “autophagy”. The search period from inception to September 2022. The searches will be re-run just before the final analyses to retrieve the most recent studies eligible for inclusion.

**Table 1:** Example of a search strategy for EMBASE.

#1	’brain ischemia’/exp
#2	’brain ischemia’:ti,ab,kw OR ’brain ischemias’:ti,ab,kw OR ’ischemia, brain’:ti,ab,kw OR ’ischemic stroke’:ti,ab,kw OR stroke:ti,ab,kw OR ’middle cerebral artery occlusion’:ti,ab,kw OR ’encephalopathy, ischemic’:ti,ab,kw OR ’ischemic encephalopathies’:ti,ab,kw OR ’cerebral ischemia’:ti,ab,kw OR ’cerebral ischemias’:ti,ab,kw OR ’ischemias, cerebral’:ti,ab,kw OR ’ischemia, cerebral’:ti,ab,kw OR mcao:ti,ab,kw OR ’brain ischaemia’:ti,ab,kw OR ’cerebral ischaemia’:ti,ab,kw OR ’cerebrovascular ischaemia’:ti,ab,kw OR ’cerebrovascular ischemia’:ti,ab,kw OR ’ischaemia cerebri’:ti,ab,kw OR ’ischaemic brain disease’:ti,ab,kw OR ’ischaemic encephalopathy’:ti,ab,kw OR ’ischemia cerebri’:ti,ab,kw OR ’ischemic brain disease’:ti,ab,kw OR ’ischemic encephalopathy’:ti,ab,kw
#3	#1 OR #2
#4	’experimental animal’/exp
#5	’animals, laboratory’:ti,ab,kw OR mice:ti,ab,kw OR rats:ti,ab,kw OR ’animal, laboratory’:ti,ab,kw OR ’laboratory animal’:ti,ab,kw OR ’laboratory animals’:ti,ab,kw OR ’experimental animals’:ti,ab,kw OR ’experimental animal’:ti,ab,kw
#6	#4 OR #5
#7	’acupuncture’/exp
#8	acupuncture:ti,ab,kw OR ’acupuncture treatment’:ti,ab,kw OR ’acupuncture treatments’:ti,ab,kw OR ’treatment, acupuncture’:ti,ab,kw OR ’acupuncture therapy’:ti,ab,kw OR ’therapy, acupuncture’:ti,ab,kw OR ’pharmacoacupuncture treatment’:ti,ab,kw OR ’treatment, pharmacoacupuncture’:ti,ab,kw OR ’pharmacoacupuncture therapy’:ti,ab,kw OR ’therapy, pharmacoacupuncture’:ti,ab,kw OR pharmacopuncture:ti,ab,kw OR acupotomy:ti,ab,kw OR acupotomies:ti,ab,kw OR electroacupuncture:ti,ab,kw OR moxabustion:ti,ab,kw OR moxibustion:ti,ab,kw OR ’scalp acupuncture’:ti,ab,kw
#9	#7 OR #8
#10	’autophagy (cellular)’/exp
#11	autophagy:ti,ab,kw OR mitophagy:ti,ab,kw OR microautophagy:ti,ab,kw OR macroautophagy:ti,ab,kw OR ’chaperone-mediated autophagy’:ti,ab,kw OR ’autophagy, cellular’:ti,ab,kw OR ’cellular autophagy’:ti,ab,kw OR autophagocytosis:ti,ab,kw OR reticulophagy:ti,ab,kw OR ’er phagy’:ti,ab,kw OR nucleophagy:ti,ab,kw OR ribophagy:ti,ab,kw OR lipophagy:ti,ab,kw OR ’cell autophagy’:ti,ab,kw
#12	#10 OR #11
#13	#3 AND #6 AND #9 AND #12

### Study selection

#### Types of studies

Original full research papers of animal studies with at least one separate control group will be included, while human studies will be excluded.

#### Types of animals

Focal cerebral infarction models of MCAO will be included without restriction on species, age or sex. Ex vivo and in vitro models will be excluded.

#### Types of intervention (treatment, control)

We will consider including the study if the intervention in the treatment group was acupuncture therapy of various common types (acupuncture, moxibustion, electroacupuncture, etc.), unless acupuncture therapy is combined with other treatments. Control groups will be those that received placebo/sham acupuncture or no treatment after model establishment.

#### Outcome measures

Studies will be included if they report neurological function scores and/or infarct volume, and autophagy was observed at the same time, while they will be excluded if no relevant outcomes are reported. The primary outcome is autophagy-related proteins/RNAs, or other autophagy-related outcomes. Infarct size, infarct volume, or neurologic deficit score is secondary outcome measures.

#### Language and publication restrictions

Reviews and editorials will be excluded. No publication date or language restrictions will be applied.

Screening will be performed in two phases, namely, initial screening based on the title and abstract, followed by a full-text screening of the eligible articles for final inclusion. In each phase, 2 observers (JXY and ZNZ) will independently assess each article. Discrepancies will be resolved through discussion or by consulting a third investigator (JXN). The searches will be rerun just before the final analyses to retrieve the most recent studies eligible for inclusion. The exclusion criteria order of priority is the same in both initial screening and full-text screening (Table 2).

**Table 2:** Exclusion criteria order of priority.

1	Reviews, editorials, conference abstracts
2	Not an in vivo animal study
3	Not a model of cerebral ischemia
4	Other focal cerebral infarction models of cerebral ischemia without MCAO
5	No separate control group, case studies
6	Intervention in treatment group was nonacupuncture therapy or acupuncture therapy combined with other treatments
7	No control group received placebo/sham acupuncture or no treatment
8	No relevant outcomes reported

### Data collection

Two reviewers (JXY and ZNZ) will independently extract data from each article, and a data extraction form made by us will be applied to data collection. We first try to extract numerical data from text, tables or figures. If these are not reported, we will extract data from graphs using Engauge Digitizer 12.1. If an outcome is measured at multiple time points, data from the time point where most treatment is executed will be included. If different outcomes of the same experiment were published in different articles, we will integrate the data. The information that we need to extract is as follows:

#### Study ID

First author, year of publication and language.

#### Study design characteristics

Experimental groups, control group(s) and the number of animals per group.

#### Animal model characteristics

Species, sex, weight, anaesthetic agent used, induction method of brain ischemia, duration of ischemia, duration of reperfusion, and sampling sites.

#### Intervention characteristics

Types of acupuncture, specific parameters of electroacupuncture (if used), acupoints, and intervention time (including time of beginning, frequency, duration and retention).

#### Outcome measures

The relative expression of autophagy-related proteins/RNAs, the number of autophagosomes per μm^2^, and any other autophagy-related outcomes will be extracted. The percentage of the infarct area and neurological deficit score are two major preclinical outcome measures for stroke pharmacotherapy studies [13], which will also be extracted. The type of all data mentioned above will be continuous. We will also extract the valuation methods.

In addition, the upregulation or downregulation of autophagy, as well as the effect of acupuncture therapy on autophagy after brain ischemia in each study, will also be collected as qualitative data.

### Data analysis

#### Risk of bias assessment

All included studies will undergo a risk of bias assessment by two reviewers (JXY and ZNZ) independently using the Systematic Review Center for Laboratory animal Experimentation (SYRCLE) risk of bias tool. Each bias parameter will be rated to have a high, low or unclear risk of bias. Any discrepancies between the two reviewers will be resolved through discussion or consultation with a third reviewer (JXN).

#### Assessment of heterogeneity

Heterogeneity will be assessed using the I² statistic before synthesizing the data. When I²<50%, the heterogeneity between studies might not be important. I²>50% will suggest the existence of heterogeneity. If I²≥75%, the heterogeneity was regarded as considerable.

#### Data synthesis and effect measure

We will apply RevMan 5.4.1 software to synthesize the data. Weighted mean differences (WMDs) or standardized mean differences (SMDs) with 95% CIs will be used to analyse continuous data (according to whether the studies use the same measurement scale or not), and risk ratios (RRs) with 95% CIs will be used to analyse dichotomous data. Random effects models more accurately reflect reality due to the heterogeneous nature of animal studies [28]. Therefore, if the included studies are sufficiently homogeneous (I^2^<50%), we will conduct a meta-analysis using a random effects model. When I²≥50%, we will try to explore and resolve the causes of heterogeneity for meta-analysis. If I²≥75% and we cannot determine the cause of heterogeneity, a descriptive analysis rather than a meta-analysis will be performed.

#### Subgroup analyses

Subgroup analyses will be conducted according to different intervention types and different types of outcomes. If necessary, subgroup analysis will also be applied to identify the cause of heterogeneity.

#### Sensitivity analyses

Sensitivity analyses will be conducted. We will conduct a merger analysis after eliminating some studies and compare the merger effect size with that before the elimination to determine the influence of the eliminated studies on the merger effect size. Methodological weaknesses, sample sizes, or missing data can also impact sensitivity analyses.

#### Correction for multiple uses of the control group

Whenever a control group served more than one experimental group, we corrected the total number of control animals in the meta-analysis by dividing the number of animals in the control group by the number of treatment groups served. Where applicable, Holm-Bonferroni correction for testing multiple subgroup analyses will be performed. If one or more subgroup analyses cannot be performed due to insufficient data, the p value will be adjusted accordingly.

#### Publication bias

Publication bias will be produced by funnel plots when more than 10 studies are included. Egger’s test and Peter’s test will be used to test the funnel plot symmetry.

#### Quality of evidence and strength of recommendations

We will apply the Grading of Recommendations, Assessment, Development, and Evaluation (GRADE) system [28] to evaluate the quality of evidence in this systematic review by two reviewers (JXY and ZNZ). Any discrepancies between the two reviewers will be resolved through discussion or consultation with a third reviewer (JXN).

## Discussion

Many in vivo studies have shown that acupuncture is effective for ischemic stroke, and the mechanism may be related to autophagy [22–25]. However, their perspectives of exploration and practical methods are not exactly the same, and the quality of evidence is limited. We intend to conduct this review since there has been no relevant systematic evaluation published before. The results of this study may help to elucidate the mechanism of acupuncture from the perspective of autophagy and provide a basis and theoretical support for the use of acupuncture in the treatment of ischemic stroke. The limitation of this review is that although no language restrictions will be applied when selecting studies, the medical databases searched will be limited to Chinese or English databases due to language barriers. Hence, we may miss some relevant articles in other languages.

## Supporting information

S1 Checklist PRISMA-P (Preferred Reporting Items for Systematic review and Meta-Analysis Protocols) 2015 checklist: Recommended items to address in a systematic review protocol. (DOC)
